# Coupling solar‐driven interfacial evaporation with forward osmosis for continuous water treatment

**DOI:** 10.1002/EXP.20220054

**Published:** 2022-07-06

**Authors:** Xiangju Song, Weichao Dong, Yajing Zhang, Hamdy Maamoun Abdel‐Ghafar, Arafat Toghan, Heqing Jiang

**Affiliations:** ^1^ Qingdao Key Laboratory of Functional Membrane Material and Membrane Technology, Qingdao Institute of Bioenergy and Bioprocess Technology Chinese Academy of Sciences Qingdao China; ^2^ University of Chinese Academy of Sciences Beijing China; ^3^ Central Metallurgical Research and Development Institute (CMRDI) Cairo Egypt; ^4^ Chemistry Department, Faculty of Science South Valley University Qena Egypt; ^5^ Chemistry Department, College of Science Imam Mohammad Ibn Saud Islamic University (IMSIU) Riyadh Saudi Arabia

**Keywords:** continuous separation, coupling system, forward osmosis, photothermal evaporation, solar energy

## Abstract

Forward osmosis (FO) driven by osmotic pressure difference has great potential in water treatment. However, it remains a challenge to maintain a steady water flux at continuous operation. Herein, a FO and photothermal evaporation (PE) coupling system (FO‐PE) based on high‐performance polyamide FO membrane and photothermal polypyrrole nano‐sponge (PPy/sponge) is developed for continuous FO separation with a steady water flux. The PE unit with a photothermal PPy/sponge floating on the surface of draw solution (DS) can continuously in situ concentrate DS by solar‐driven interfacial water evaporation, which effectively offsets the dilution effect due to the injected water from FO unit. A good balance between the permeated water in FO and the evaporated water in PE can be established by coordinately regulating the initial concentration of DS and light intensity. As a consequence, the polyamide FO membrane exhibits a steady water flux of 11.7 L m^–2^ h^–1^ over time under FO coupling PE condition, effectively alleviating the decline in water flux under FO alone. Additionally, it shows a low reverse salt flux of 3 g m^–2^ h^–1^. The FO‐PE coupling system utilizing clean and renewable solar energy to achieve a continuous FO separation is significantly meaningful for practical applications.

## Graphical abstract

A forward osmosis and photothermal evaporation coupling system (FO‐PE) based on high‐performance polyamide FO membrane and photothermal polypyrrole nano‐Sponge (PPy/Sponge) is developed for continuous FO separation with a steady water flux. The FO‐PE coupling system utilizing clean and renewable solar energy to achieve efficient water treatment is significantly meaningful for practical applications.

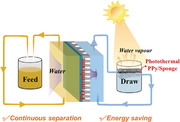

## 1 INTRODUCTION

With the rapid population growth and industrial expansion, freshwater scarcity has been a global issue, which seriously threatens human life and social development.^[^ ^1^, ^2^, ^3^ ^]^ To address the water crisis, numerous efforts have been dedicated to developing water purification technologies. Among the available options, membrane‐based separation technology is considered to be a powerful tool owing to its high water recovery, energy efficiency, and ease to integrate with other technologies.^[^ ^4^, ^5^, ^6^, ^7^ ^]^ As a special role in membrane families, forward osmosis (FO), can realize the efficient separation of water and solutes depending on the osmotic pressure difference without external pressure input.^[^ ^5^ ^]^ It has been widely recognized as an energy‐efficient technology, and has extended its application in juice concentration and pharmaceutical processing.^[^ ^8^, ^9^, ^10^, ^11^, ^12^ ^]^ However, in practical operations, the water flux and separation efficiency decrease significantly over time because water continuously diffuses from a feed solution (FS with a low osmotic pressure) through FO membrane to a draw solution (DS with a high osmotic pressure),^[^ ^13^ ^]^ decreasing the osmotic driving force. Although periodical replacement of DS or concentrating DS after FO process can solve the aforementioned issue, they will arouse high energy consumption and complex operation.

Recently, some studies reported that integrating nanofiltration (NF) or membrane distillation (MD) with FO could achieve DS concentration and recycle.^[^ ^14^, ^15^, ^16^ ^]^ For example, Corzo's group developed a FO‐NF technology, which realized a steady water permeability during long‐term operation, and reduced the investment cost arising from DS replacement.^[^ ^14^ ^]^ Hu et al. employed an integrated FO‐MD technology for continuous juice concentration.^[^ ^15^ ^]^ They used the upstream FO process to concentrate apple juice, and the downstream MD to recover draw solutes via transforming water into steam under high temperature. Benefiting from the unchanged osmotic pressure of DS, the FO‐MD hybrid technology achieved a steady water flux in FO process. Nevertheless, NF and MD are driven by external pressure and vapor pressure difference, respectively, which lead to high energy consumption despite the upstream FO process is energy‐saving. Some researchers have also reported that in integrated systems such as FO‐MD, FO‐reverse osmosis (RO), and some others, the proportion of energy consumed by regenerating DS is relatively high.^[^ ^17^, ^18^, ^19^ ^]^ In this regard, it is highly desirable to develop a new downstream process by using renewable energy to concentrate DS and thus reduce the energy consumption of the whole separation system.

Photothermal water evaporation, utilizing sustainable solar energy to concentrate solution, demonstrates distinct advantages compared to the energy‐intensive MD and NF. Despite the fact that some trials have been tried via directly heating bulk DS using solar energy, the concentration efficiency was extremely low due to the poor light‐harvesting and the serious heat loss.^[^ ^20^, ^21^ ^]^ By contrast, solar‐driven interfacial water evaporation based on photothermal membranes or solar absorbers shows prominent features because it concentrates thermal energy at the air/liquid interface, significantly reduces heat loss, and hence improves water evaporation efficiency.^[^ ^22^, ^23^, ^24^, ^25^ ^]^ Based on the aforementioned features, the solar‐driven interfacial water evaporation is a good energy‐efficient choice to substitute the NF/RO and MD for concentrating DS in FO process. In previous work, we have attempted to concentrate sucrose DS using photothermal water evaporation dependent on functional‐multiwalled carbon nanotubes (f‐MWCNTs) based photothermal membrane, which demonstrated a good result.^[^ ^26^ ^]^ However, the independent concentration process after FO separation is time‐consuming. Supposing integrating FO with photothermal evaporation (PE) into one process, the coupling system may not only in situ concentrate DS, but also realize the continuous separation in FO process by matching the FO and PE processes. While, there is still no relevant research via coupling FO with solar‐driven interfacial evaporation to achieve high‐efficient and sustainable FO separation.

Herein, for the first time, we developed a FO‐PE coupling system comprehensively utilizing the advantages of FO and PE, which improves the separation efficiency of FO process. A thin‐film composite (TFC) polyamide membrane with high permeance and selectivity was used as FO membrane. Meanwhile, considering the influence of the microstructure and physicochemical properties of light absorbers on light absorption and light‐to‐heat conversion properties, as well as water evaporation efficiency, a polypyrrole (PPy) modified nano‐sponge (PPy/sponge) with large and interconnected pores was successfully constructed to maintain high water evaporation performance in PE process. By integrating FO and PE processes into one system, and regulating the matching between water permeation amount and water evaporation amount per unit time in FO and PE processes, respectively, FO separation process with a steady water flux can run continuously over time. This pioneering design of integrating FO with PE process provides a new route to establish energy‐saving separation systems by sustainable solar energy for high efficient wastewater treatment and seawater desalination.

## 2 RESULTS AND DISCUSSION

### 2.1 **The structures and properties of polyamide membrane and photothermal PPy/**s**ponge**

**FIGURE 1:**
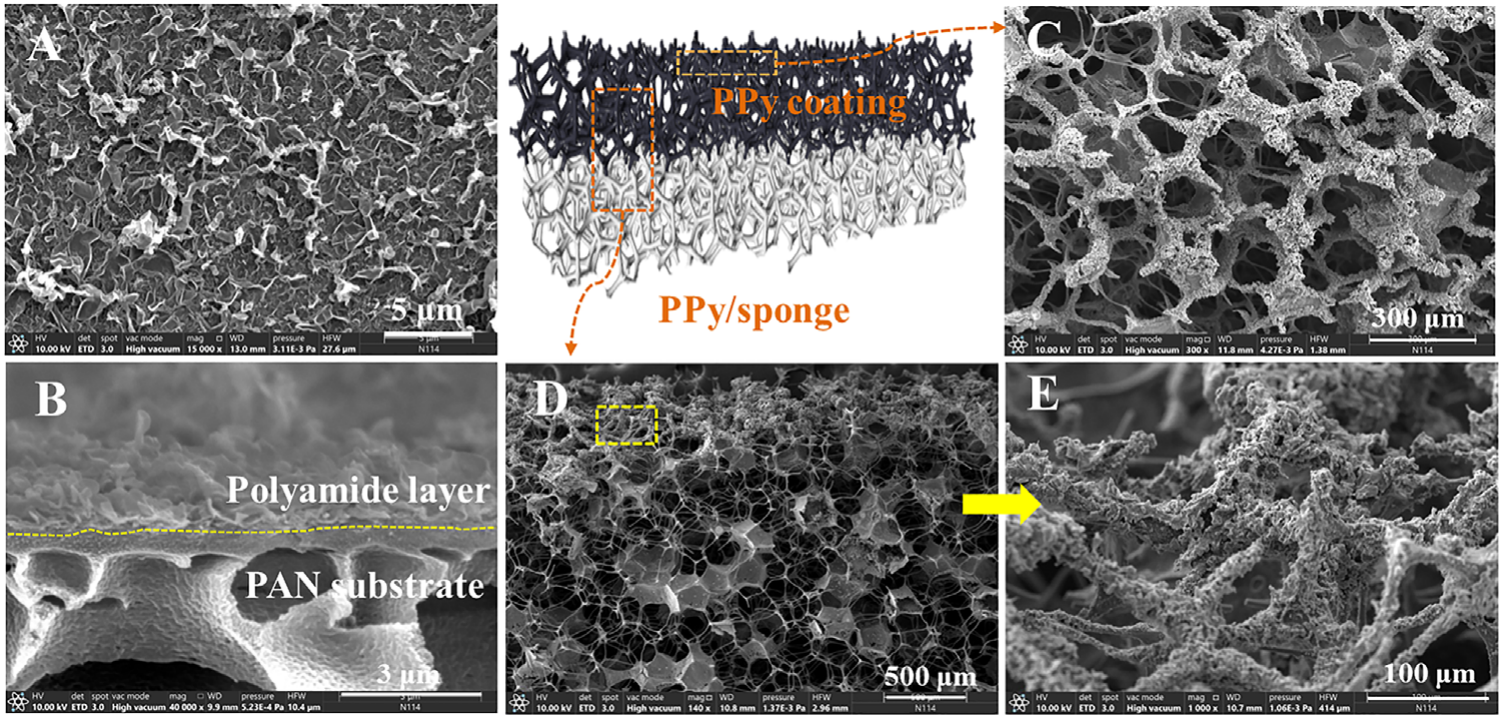
The SEM images of (A,B) TFC polyamide FO membrane and (C,D) photothermal PPy/sponge, respectively. (E) High‐magnification image recorded from the marked zone in (D)

To investigate the morphologies and microstructures of the polyamide FO membrane and photothermal PPy/sponge, scanning electron microscopy (SEM) characterization was carried out. The polyamide membrane was fabricated via a typical interfacial polymerization between *m*‐phenylene diamine (MPD) and trimesoyl chloride (TMC) upon support layer. Figure 1A,B shows an ultrathin active layer with leaf‐like structures formed on porous substrate. The architectural features are mainly attributed to the interfacial polymerization reaction far from thermodynamic equilibrium.^[^ ^27^ ^]^ Specifically, the violent reaction between MPD and TMC generates a great deal of heat, which promotes the rapid migration of MPD to the organic phase, and leads to the growth of polyamide perpendicular to the reaction interface, eventually giving rise to roughly crumpled structures. Generally, the rough surface will expand the effective contact area between water and membrane, tending to generate a high water flux.^[^ ^28^, ^29^ ^]^ Besides, the structure of photothermal PPy/sponge in PE unit was also investigated. As shown in the SEM images (Figure 1C,D), the PPy/sponge has a large and pore‐interconnected network structure either the surface or cross‐section. After modification with PPy, the photothermal PPy/sponge still remains its original porous structures, and the PPy is only deposited onto the framework. Such interconnected macropores play a vital role in water and vapor transport, thereby contributing to the efficient water evaporation. The PPy coating located on the upper side of the sponge (Figure 1E) is beneficial to capture light and convert it into heat, facilitating water evaporation, thus concentrating DS continuously.

**FIGURE 2:**
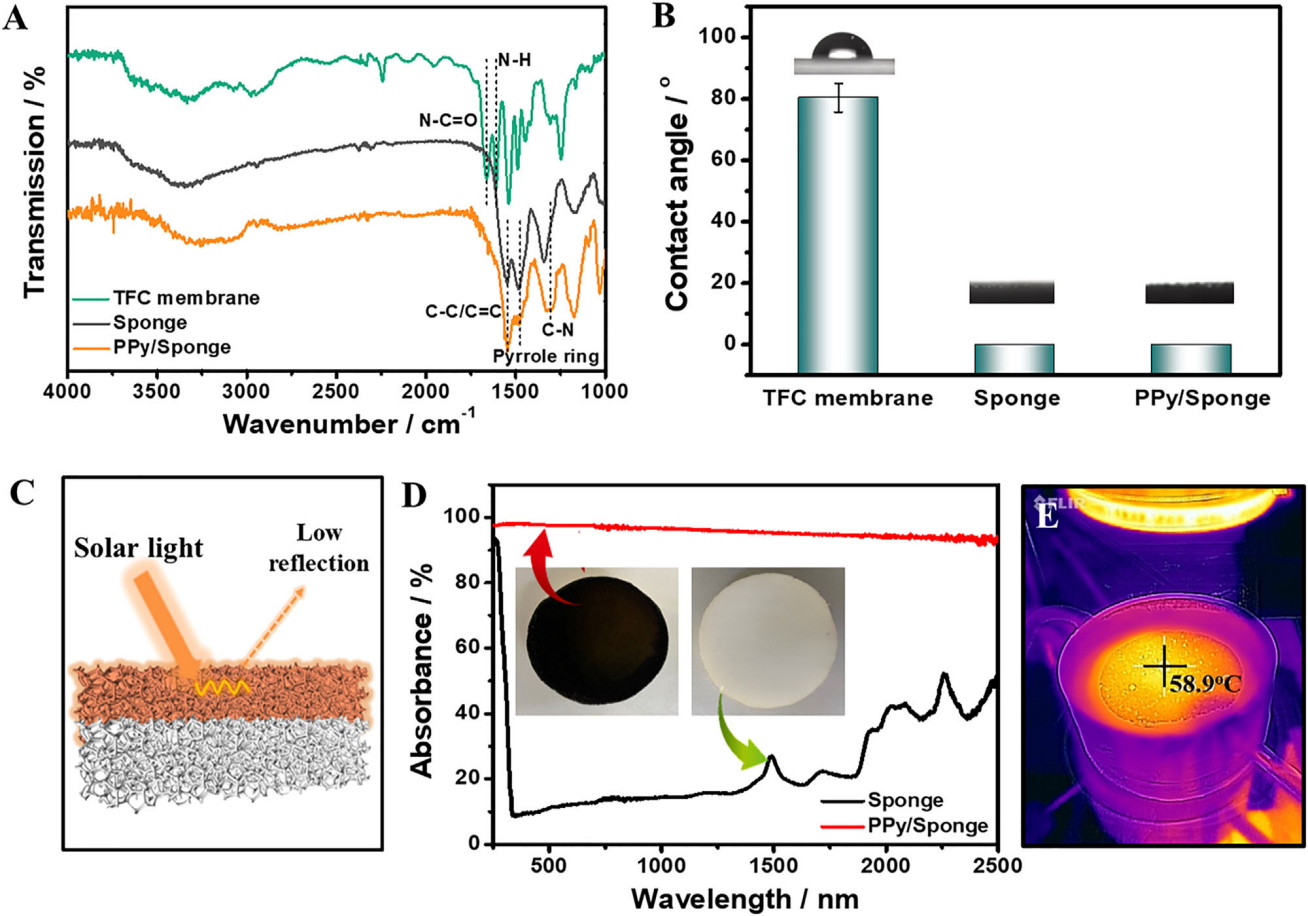
(A) FTIR spectra and (B) water contact angle of the TFC membrane, the control nano‐sponge, and PPy/sponge. (C) Schematic illustration of PPy/sponge with large network pores to decrease the light reflection. (D) The absorbance spectra of the nano‐sponge and PPy/sponge. (E) IR images of the PPy/sponge

The chemical compositions of TFC polyamide FO membrane and photothermal PPy/sponge were characterized by Fourier transform infrared (FTIR) spectroscopy. Figure 2A shows the TFC membrane obtains the typical peaks of polyamide at 1660, 1610, and 1540 cm^–1^, representing C═O (amide I), N─H stretching vibration (amide II), and in‐plane N─H bending and C─N stretching vibrations (amide II), respectively.^[^ ^30^, ^31^, ^32^ ^]^ Moreover, the band located at 1725 cm^–1^ is associated with the C═O stretching vibration of carboxyl originating from the hydrolysis of unreacted acyl‐chloride groups.^[^ ^33^, ^34^ ^]^ These functional groups can improve membrane hydrophilicity.^[^ ^35^, ^36^ ^]^ Hydrophilic surface can capture more water molecules and enhance water flux. The TFC membrane exhibits a contact angle of 80^°^ (Figure 2B). For the photothermal sponge, it obtains several peaks at 1541, 1472, and 1282 cm^–1^, corresponding to C─C/C═C stretching, pyrrole ring, and C─N stretching vibration, respectively,^[^ ^24^, ^37^ ^]^ which indicates the successful deposition of PPy on nano‐sponge.

**FIGURE 3:**
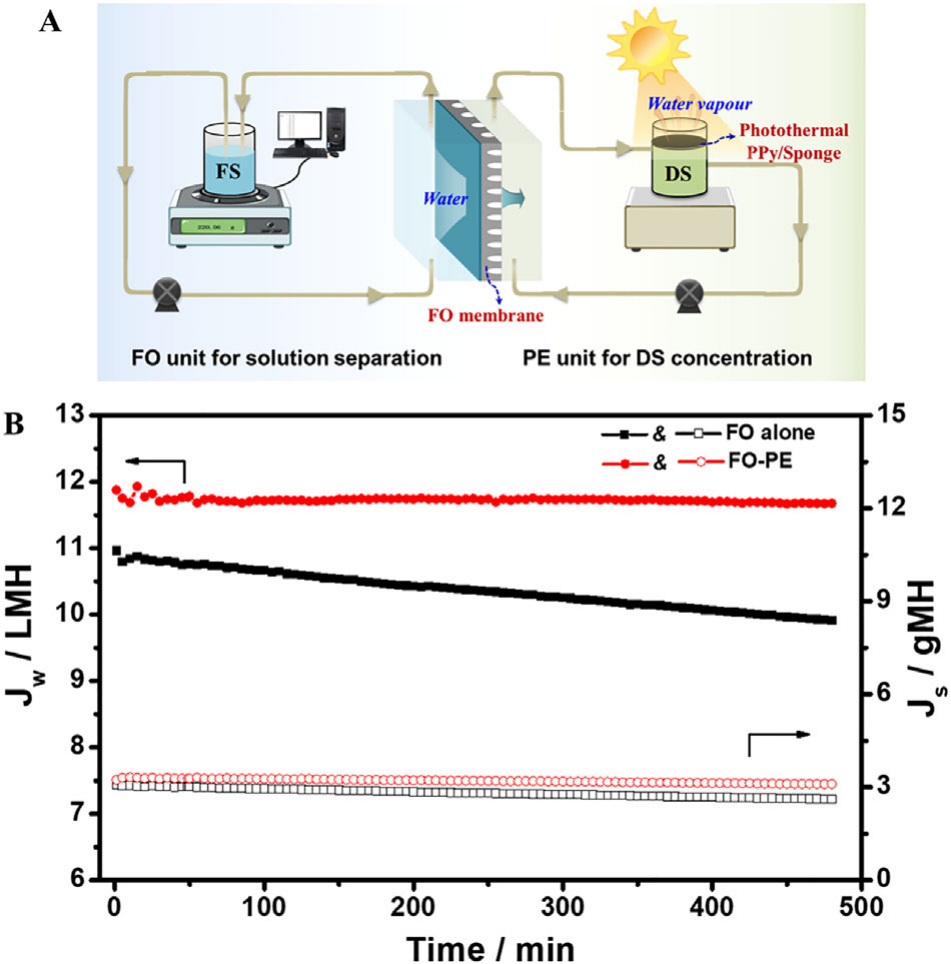
(A) Schematic diagram of FO‐PE coupling system. (B) Separation performance in terms of water flux and reverse salt flux of TFC polyamide membrane as a function of time in FO alone and FO‐PE systems with light intensity of 10 kW m^–2^, respectively.

To further evaluate light harvesting ability, UV‐vis‐NIR spectrometer was applied to characterize the light reflectance, transmittance, and absorbance of nano‐sponge. The results (Figure S1) show in comparison with the pristine nano‐sponge, the PPy/sponge possesses an extremely low reflection and transmission close to zero over the whole solar spectra range (250–2500 nm). The light absorption capacity of the PPy/sponge is higher than 90% (absorption = 100% – transmittance – reflectance), demonstrating an excellent light harvesting capacity (Figure 2D). Meanwhile, the PPy/sponge exhibits a superior light‐to‐heat conversion property, it reaches a steady temperature about 58.4 ± 1.8°C within 3 min (Figure S2) under the FO‐PE condition (Figure 2E). The excellent light harvesting and light‐to‐heat conversion property mainly benefit from the particular microstructure and properties of PPy/sponge. On one hand, the intrinsic dark color of PPy contributes to light absorption at a broad‐spectrum. On the other hand, the large network pores make light undergo multiple reflection inside the interconnected pores, therefore greatly reducing the diffuse reflectance and improving light harvesting (Figure 2C).^[^ ^24^ ^]^ As a result, it will facilitate the fast water evaporation from the interface between PPy/sponge and air.^[^ ^38^, ^39^ ^]^ Another important factor that affects water evaporation is water transport in the bulk PPy/Spong, which is determined by the wettability of the photothermal PPy/sponge. Therefore, the surface wettability of the PPy/sponge was studied. As shown in Figure 3B, both the pristine sponge and PPy/sponge exhibit good water hydroplilicity, water spreads quickly once the water droplet makes contact with the sponge, which indicates the nano‐sponge is a able water transport medium. Owing to the good water hydrophilicity and light‐to‐heat conversion properties, the PPy/sponge holds great promise for improving water evaporation performance. Namely, it can effectively concentrate DS using renewable solar energy, and decrease the whole energy consumption of the FO‐PE coupling system.

### 2.2 Separation performance of polyamide membrane under FO‐PE condition

The FO separation is achieved by water diffusion through FO membrane from the low‐concentrated FS to high‐concentrated DS. With time extension, the DS is diluted gradually, decreasing the osmatic driving force, and further deteriorating the water flux. Here, a photothermal evaporation unit was constructed to in situ concentrate DS via water evaporating from the interface between photothermal PPy/sponge and air under light illumination. The schematic diagram of FO‐PE coupling system is illustrated in Figure 3A.

To demonstrate the enhancement effect of solar‐driven interfacial evaporation on FO, the separation performance of TFC polyamide membrane under FO alone and FO‐PE coupling system was performed. Figure 3B presents the water flux decreasing obviously over time under FO alone condition, which is resulted from the gradually decreased osmotic pressure difference due to the continuous water injection in DS. The reverse salt flux maintains at 3 g m^–2^ h^–1^ (gMH) benefiting from the good selectivity of TFC membrane. By contrast, an almost steady water flux of 11.7 L m^–2^ h^–1^ (LMH) is obtained with the assistance of PE unit. The main reason is the coupling effect of FO and PE. Specifically, water diffusion from feed to DS decreases the osmatic pressure gradually, producing a negative effect on water flux. At the same time, water gradually escapes from the DS and the salt retained in the PPy/sponge returns to the bulk solution along with the cycle of DS, elevating the osmatic pressure of DS. Based upon the counter effect of water injection and water evaporation, a relatively stable osmatic pressure difference is obtained, thus maintaining the stability of water flux. Meanwhile, the reverse salt flux is almost unchanged. This is predictable because the heat generated by solar energy concentrates at the interface of PPy/sponge and air, the temperature of bulk DS is about 27 ± 1°C, slightly higher than that of the nascent solution (23 ± 0.5°C), which may contribute to a slightly elevated water flux in FO‐PE system. Figure S3A shows the protuberant microstructure on polyamide membrane after the test becomes less obvious compared with the fresh membrane (Figure S3B). There is no defect observed on membrane surface. Furthermore, the polyamide membrane shows a nearly constant reverse salt flux during the test. The results suggest the slightly increased temperature of DS does not destroy the perm‐selectivity of the membrane. Additionally, the FO‐PE coupling system exhibits a good stability in two consecutive cycle tests (Figure S4). The results indicate the combination of FO and PE is a promising strategy to achieve a continuous FO separation with a steady water permeance, which utilizes clean and renewable solar energy to offset chemical potential energy, effectively decreasing energy consumption.

In FO‐PE coupling system, the relation between water flux in FO and water evaporation rate in PE is vital for the final separation performance and the continuity of the whole system. Achieving a good matching between the permeated water amount and evaporated water amount per unit time in FO and PE process, respectively, can reach a constant osmotic pressure of DS, thereby generating a continuous and steady FO separation. The water flux in FO is mainly dominated by osmotic pressure difference, which is determined by the osmatic pressure of the FS and DS on both sides of FO membrane. In PE process, for a certain photothermal absorber, the light intensity determines the water evaporation performance, a high light intensity generates a high evaporation rate. Thus, the influences of DS concentration and light intensity on the separation performance of FO‐PE system were systematically investigated.

**FIGURE 4:**
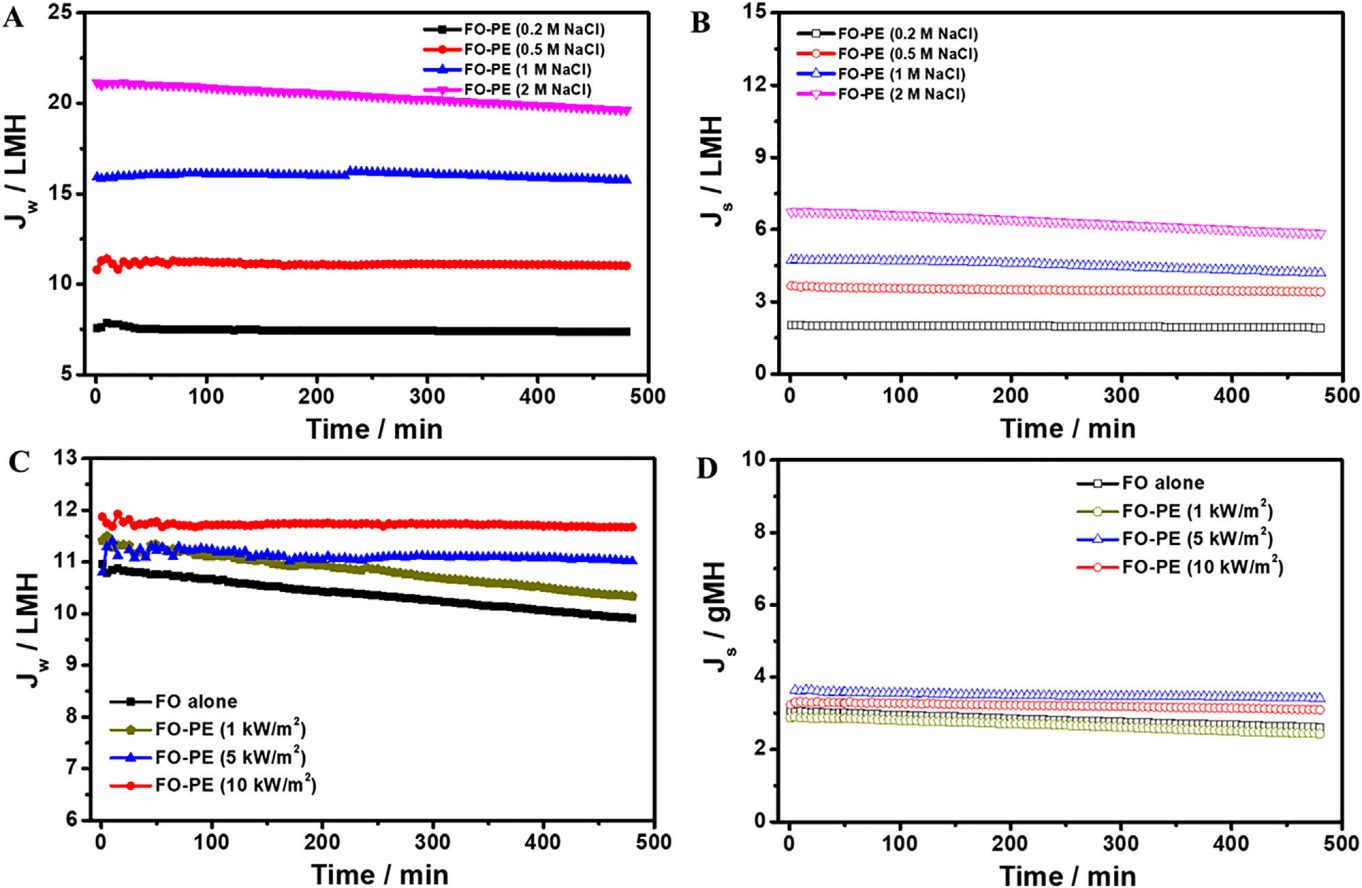
The influence of DS concentration on (A) water flux and (B) reverse salt flux with light intensity of 5 kW m^–2^. (C) Water flux and (D) reverse salt flux with DS concentration of 0.5 mol L^–1^ under different light intensities

Figure 4A illustrates water flux increases with increasing DS concentration, which is attributed to a high DS concentration generating a high osmotic pressure, and accelerating water transport through the membrane. It is worth noting that water flux obviously decreases with time extension when 2 mol L^–1^ NaCl solution is served as DS. The reason is that the evaporated water amount under light intensity of 5 kW m^–2^ in PE process cannot completely offset the permeated water in FO process. As a result, the DS concentration is still falling, and thus water flux gradually decreases over time. Higher DS concentration leads to higher reverse salt transport through FO membrane, hence the reverse salt flux displays a slightly increase with increasing DS concentration (Figure 4B). The results demonstrate that the DS concentration has an impact on the continuity of FO separation performance through employing FO‐PE coupling system. Subsequently, another important factor, light intensity was also adjusted from 0 to 10 kW m^–2^ to investigate its influence on FO separation performance.

As shown in Figure 4C, water flux shows a decreasing tendency when using a relative weak light (such as 1 kW m^–2^) to illuminate the photothermal PPy/sponge. With increasing the light intensity, a steady water flux is obtained. Additionally, the polyamide membrane obtains a relatively invariable reverse salt flux of 3∼3.5 gMH without being affected by light intensity (Figure 4D). The slight increase in reverse salt flux is probably attributed to the slightly elevated temperature of DS at high light intensity. Generally, the thermal motion of salt ions is intensified at high temperature, which promotes the diffusion of salt through polyamide membrane. Therefore, a slightly increased reverse salt flux is obtained at high light intensity. The results indicate the balance between the permeated water amount in FO process and evaporated water amount in PE process is a critical condition to keep a continuous FO separation. Although manipulation of the DS concentration or light intensity can realize the above objective, the former not only generates a high reverse salt flux, but also expends more chemical resources. By contrast, the latter has advantages because it maintains an invariable reverse salt flux, more importantly, obtains a continuous FO separation by utilizing sustainable solar energy. Besides, improvement of water evaporation rate via optimizing the structure and composition of photothermal absorbers/membranes can also intensify the efficiency of the FO‐PE system. Many photothermal membranes with high evaporation performance are promising candidates in this coupling system.

**FIGURE 5:**
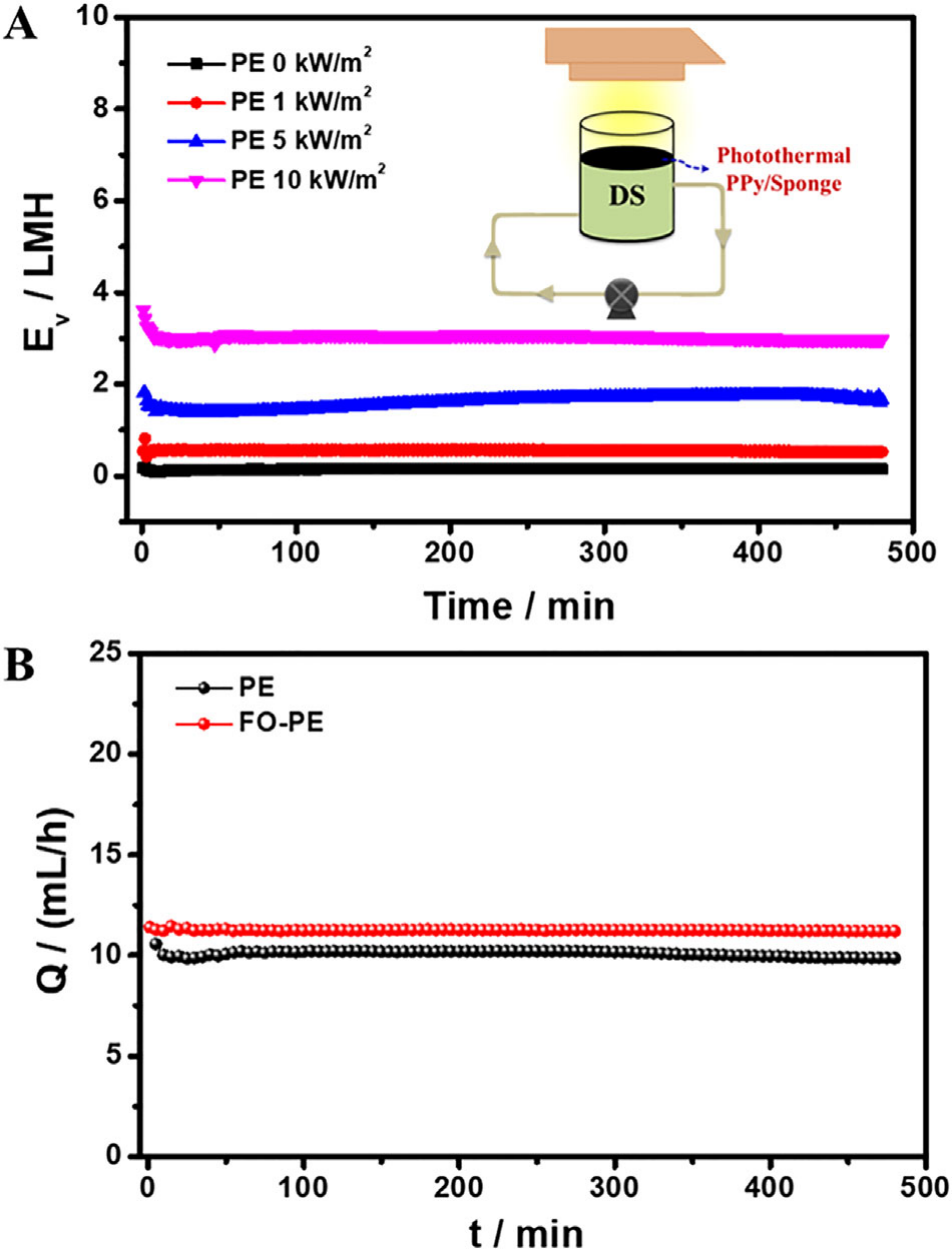
(A) Water evaporation rate of photothermal PPy/sponge under different light intensities. (B) The comparison of water evaporation amount and water permeation amount per unit time in PE and FO‐PE, respectively

To deeply investigate the relations between PE and FO unit, the evaporated water amount and permeated water amount per unit time in PE and FO‐PE were compared, respectively. Figure 5A shows the evaporation rate is around 3 LMH under the light illumination of 10 kW m^–2^. The effective evaporation area is 33.2 cm^2^, the evaporation amount is about 10 mL h^–1^ (evaporation amount = evaporation rate × evaporation area). For FO process with the assistance of PE, the water flux is around 11.7 LMH, the area of FO membrane is 9.6 cm^2^, and thus the water permeation amount is about 11.2 mL h^–1^, which is very close to water evaporation amount in PE process (Figure 5B). As a result, a steady water flux is achieved with the assistance of solar‐driven water evaporation process. To reach high energy efficiency, on one hand, installing a closed glass dome on PE unit can decrease heat convection as well as recover latent heat from condensed water. On the other hand, placing a thermal insulating layer beneath the photothermal PPy/sponge to decrease heat conduction from photothermal absorber to bulk solution is also an available strategy.

Considering the existence of organic/inorganic substances in raw water affecting the separation efficiency of the coupling system, we investigate the influence of the ordinary organic pollutant (bovine serum albumin, BSA) on the separation performance of FO‐PE system. The experiment was performed by using 500 mg/L of BSA as FS, and 0.5 mol/L of NaCl as DS. UV−Vis spectrophotometer was applied to analyze the BSA concentration in permeate solution and calculate the rejection. Figure S5A shows water flux increases slightly over time. Affected by BSA contaminant, the permeated water amount in FO unit is lower than the evaporated water amount in PE unit due to BSA adsorbing on FO membrane surface. As a result, the osmotic driving force increases, which results in a gradually increased water flux. Additionally, the polyamide FO membrane obtains an extremely high rejection of 99.4% to BSA (Figure S5B). The results suggest the potential of FO‐PE coupling system in wastewater treatment.

## 3 CONCLUSIONS

In conclusion, we have developed a FO‐PE coupling system based on high‐performance TFC polyamide FO membrane and photothermal PPy/sponge, which realized a continuous FO separation with a steady water flux. In individual FO separation, the water flux obviously decreases with time extension due to the gradually decreased osmatic driving force. With the assistance of PE process, the dilution effect of DS arising from the injection of water in FO process can be effectively counteracted by the interfacial water evaporation driven by solar energy. By adjusting DS concentration and light intensity, the permeated water amount in FO process can balance with the evaporated water amount in PE process, thereby maintaining an invariable osmotic pressure of DS. As a result, a steady water flux of 11.7 LMH was obtained. The design of FO‐PE coupling system can achieve high efficient FO separation by utilizing the clean and renewable solar energy, effectively decreasing energy consumption, which holds a great potential in a continuous and long‐term FO desalination.

## 4 EXPERIMENTAL SECTION

### 4.1 **Fabrication of polyamide membrane and photothermal PPy/**s**ponge**

**FIGURE 6:**
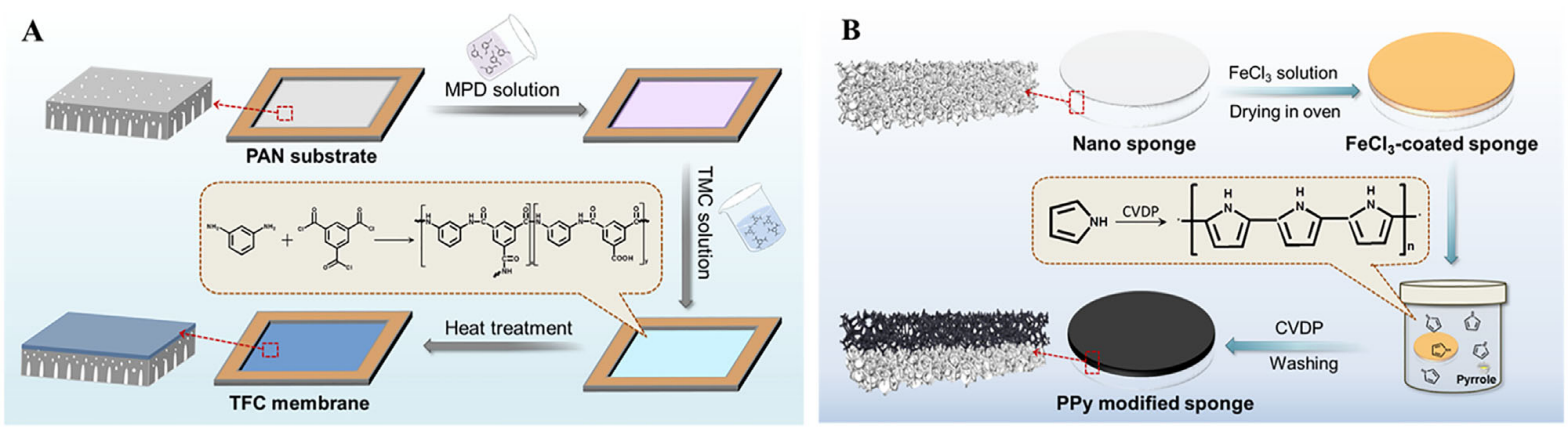
The fabrication procedures of (A) TFC polyamide FO membrane and (B) photothermal PPy/sponge

The TFC polyamide membrane was fabricated via an interfacial polymerization (IP) technique, shown in Figure 6A. Specifically, the PAN substrate was first immersed into a MPD (2 wt%) aqueous solution containing 0.15 wt% sodium dodecyl sulfate for 2 min. Afterward, the extra amine solution was drained off and the membrane was dried in air. Then, the resulting membrane was contacted with 0.1 w/v% of TMC/*n*‐hexane solution for 1 min to motivate the reaction between MPD and TMC. Subsequently, the nascent polyamide membrane was heated at 80°C for 5 min for further reaction. The resultant membrane was then rinsed and soaked in deionized water before FO test.

Figure 6B illustrates the fabrication procedure of photothermal nano‐sponge. The control nano‐sponge with a diameter of 6.5 cm was first immersed in 0.5 mol/L FeCl_3_ solution (ethanol: DI water = 4: 1) for 5 min. Thereafter, the FeCl_3_‐containing nano‐sponge was dried at 50°C for 3 h. Subsequently, the resultant sponge was sealed into a container with 50 µl of pyrrole at 50°C for 2 h. During the chemical vapor deposition polymerization, pyrrole occurred self‐polymerization under the oxidation of FeCl_3_, which produced a dark PPy coating on nano‐sponge. The resultant photothermal sponge designated as PPy/sponge was thoroughly washed with ethanol and deionized water with the assistance of sonication, followed by drying overnight at 50°C.

### 4.2 Characterizations

The morphologies of the samples were captured by a SEM (Thermo Scientific Prisma E). The functional groups of the polyamide membrane and photothermal PPy/sponge were investigated by FTIR spectrometer (Nicolet iS10). The surface wettability was examined with a contact angle analyzer (JC2000DM). The diffuse reflectance and transmittance were recorded on a UV–vis‐NIR spectro‐photometer (Hitachi U‐4100) with wavelength coverage from 250 to 2500 nm. The surface temperature was recorded by a FLIR One infrared camera.

### 4.3 Separation performance test of the FO‐PE coupling system

The separation performance of the polyamide membrane was evaluated by a coupling system, consisting of FO and PE units. In FO unit, DI water and 0.5 mol L^–1^ of NaCl solution were used as feed solution (FS) and DS, respectively, which were circulated on the both sides of the polyamide membrane by two gear pumps. During the test, the dense polyamide active layer of TFC membrane faced to FS. In PE unit, the photothermal PPy/sponge was placed onto the surface of DS, which was irradiated by a solar simulation xenon lamp (CELPE300L‐3A, China) with light intensity of 10 kW m^–2^ and wavelength from 280 to 2500 nm. The FO separation performance was measured using the coupling system. The water flux (*J*_v_, L m^–2^ h^–1^, abbreviated as LMH) was calculated according to Equation (1):^[^ ^30^, ^40^ ^]^

(1) $$J_{v}=\frac{\Delta m}{A\;\times \;\Delta t\;\times \;\rho_{0}}$$

where Δ*m* (g) is the weight change over a period of Δ*t* (h). *A* (m^2^) is the membrane area (9.6 cm^2^), and *ρ*_0_ is the water density at room temperature.

Reverse salt flux (*J*_s_, g m^–2^ h^–1^, abbreviated as gMH) was calculated with Equation (2):^[^ ^30^, ^40^ ^]^

(2) $$J_{s}=\frac{C_{t}V_{t}\;-\;C_{0}V_{0}}{A_{m}\;\times \;\Delta t}$$

where *C*_0_ (g L^–1^) and *V*_0_ (L) are the initial concentration and volume of the FS, respectively. The *C*_t_ (g L^–1^) and *V*_t_ (L) are the concentration and volume of the FS over an interval of Δ*t* (h), respectively. Herein, the salt concentration was recorded according to the conductivity.

For comparison, the separation performance of the TFC polyamide membrane without PE assistance (FO alone) was also evaluated. To check the influence of photothermal water evaporation on FO performance, the water evaporation performance of the PPy/sponge was measured with light intensity varying from 0 to 10 kW m^–2^. Specifically, the PPy/sponge was placed on the surface of NaCl solution (0.5 mol L^–1^), and a gear pump was used to circulate the solution. The weight of FS was recorded by an analytical balance connected to computer. The evaporation rate (*E*_v_, L m^–2^ h^–1^) was calculated by Equation (3):^[^ ^23^ ^]^

(3) $$E_{V}=\frac{\Delta m}{\rho_{0}\times \;S\;\times \;\Delta t}$$

where Δ*m* (g) is the weight change over a period of Δ*t* (h), *S* (m^2^) is the evaporation area (33.2 cm^2^ in this work).

Moreover, to further investigate the relations between water flux in FO and water evaporation amount in PE, the separation performance of polyamide membrane was measured by tuning the DS concentration (0.2, 0.5, 1.0, and 2.0 mol L^–1^) and light intensity (1, 5, and 10 kW m^–2^).

## CONFLICT OF INTEREST

The authors declare no conflict of interest.

## Supporting information



Figure S1. (A) The diffuse reflection and (B) transmission of the pristine nano‐sponge and photothermal PPy/sponge.
Figure S2. The surface temperature of PPy/sponge as a function of time under the FO‐PE condition.
Figure S3. The surface morphology of polyamide FO membrane (A) after and (B) before the FO‐PE test.
Figure S4. The separation performance of FO‐PE coupling system in two consecutive cycle tests.
Figure S5. Separation performance of the polyamide FO membrane under FO‐PE condition.

## Data Availability

The data that support the findings of this study are available from the corresponding author upon reasonable request.
