# Supplementary material for: Coupling solar‐driven interfacial evaporation with forward osmosis for continuous water treatment
Source: Exploration (Beijing). 2022 Jul 6;2(4):20220054. doi: 10.1002/EXP.20220054 (PMC10191015; doi:10.1002/EXP.20220054)
Supplement: Supplementary file 1 — Figure S1. (A) The diffuse reflection and (B) transmission of the pristine nano‐sponge and photothermal PPy/sponge. Figure S2. The surface temperature of PPy/sponge as a function of time under the FO‐PE condition. Figure S3. The surface morphology of polyamide FO membrane (A) after and (B) before the FO‐PE test. Figure S4. The separation performance of FO‐PE coupling system in two consecutive cycle tests. Figure S5. Separation performance of the polyamide FO membrane under FO‐PE condition. [file EXP2-2-20220054-s001.docx]

**Coupling solar-driven interfacial evaporation with forward osmosis for continuous water treatment**

Xiangju Song^1^, Weichao Dong^1,2^, Yajing Zhang^1,2^, Hamdy Maamoun Abdel-Ghafar^1,3^, Arafat Toghan*^4,5^, Heqing Jiang*^1^

*^1^Qingdao Key Laboratory of Functional Membrane Material and Membrane Technology, Qingdao Institute of Bioenergy and Bioprocess Technology, Chinese Academy of Sciences, Qingdao 266101, China.*

*^2^* *University of Chinese Academy of Sciences, Beijing 100049, China*

*^3^Central Metallurgical Research and Development Institute (CMRDI), P.O. Box: 87 Helwan, Cairo 11421, Egypt.*

*^4^Chemistry Department, Faculty of Science, South Valley University, Qena 83523, Egypt.*

*^5^Chemistry Department, College of Science, Imam Mohammad Ibn Saud Islamic University (IMSIU), Riyadh 11623, Saudi Arabia.*

*Corresponding author. Tel.: +86 532 80662716; Fax: +86 532 80662716.

E-mail address: [jianghq@qibebt.ac.cn](mailto:jianghq@qibebt.ac.cn) (H. Jiang), arafat.toghan@yahoo.com (A. Toghan).

1. Materials

Polyacrylonitrile ultrafiltration membrane with MWCO of 50,000 Da was obtained from Rising Sun Membrane Technology Co. Ltd. M-phenylenediamine (MPD, 99.5%), sodium dodecyl sulfate (SDS, >92.5%) and pyrrole were purchased from Aladdin Chemical Co., Ltd. Trimesoyl chloride (TMC, > 98%) was obtained from TCI Co., Ltd. N-hexane (≥97% ) was obtained from Tianjin Fuyu Fine Chemical Co., Ltd. Ferric chloride hexahydrate (FeCl_3_.6H_2_O), NaCl and ethanol were obtained from Sinopharm Chemical Reagent Co., Ltd.


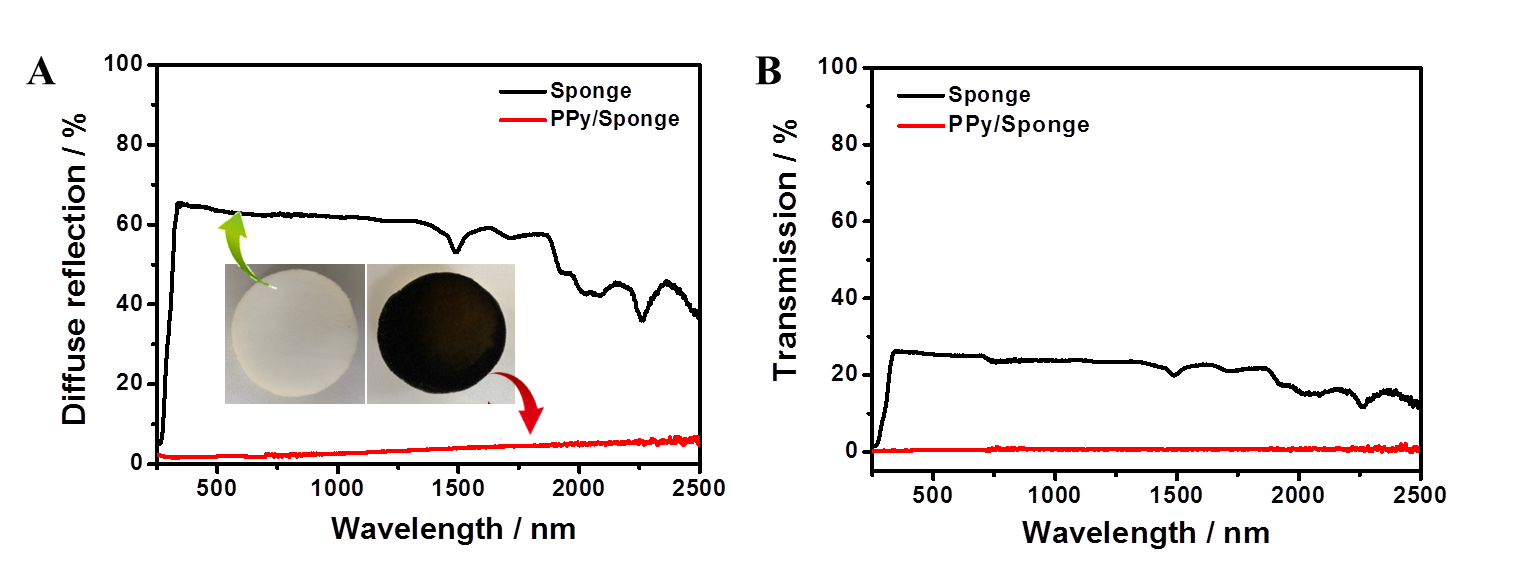


**Figure S1.** The diffuse reflection (A) and transmission (B) of the pristine nano-sponge and photothermal PPy/Sponge.


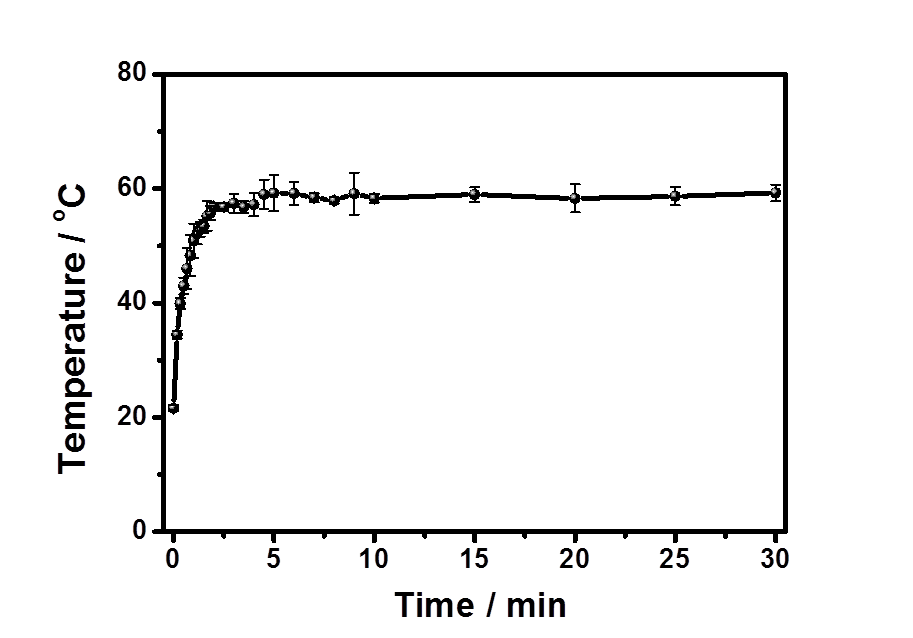


Figure S2. The surface temperature of PPy/Sponge as a function of time under the FO-PE condition.


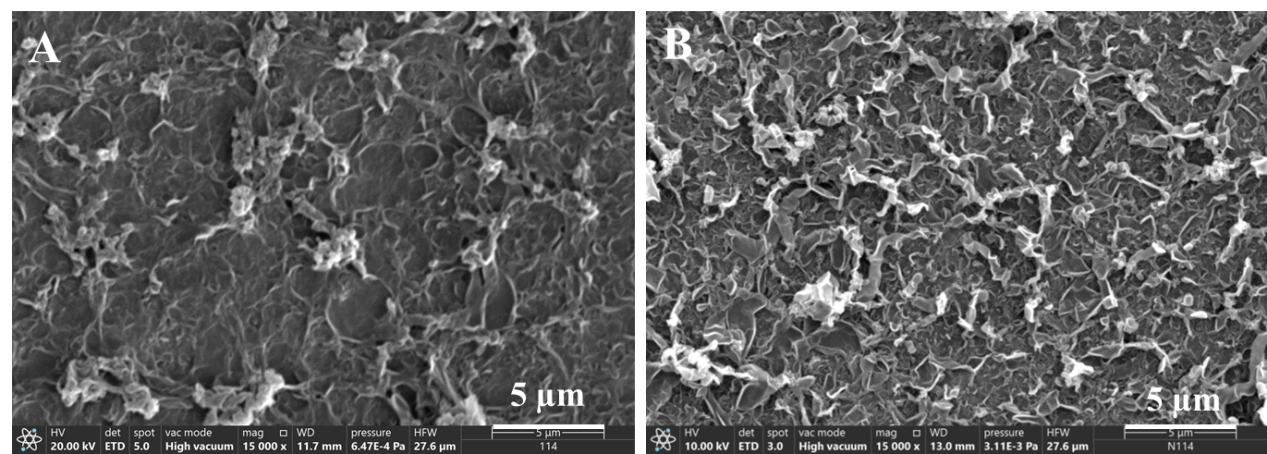


Figure S3. The surface morphology of polyamide FO membrane (A) after and (B) before the FO-PE test.


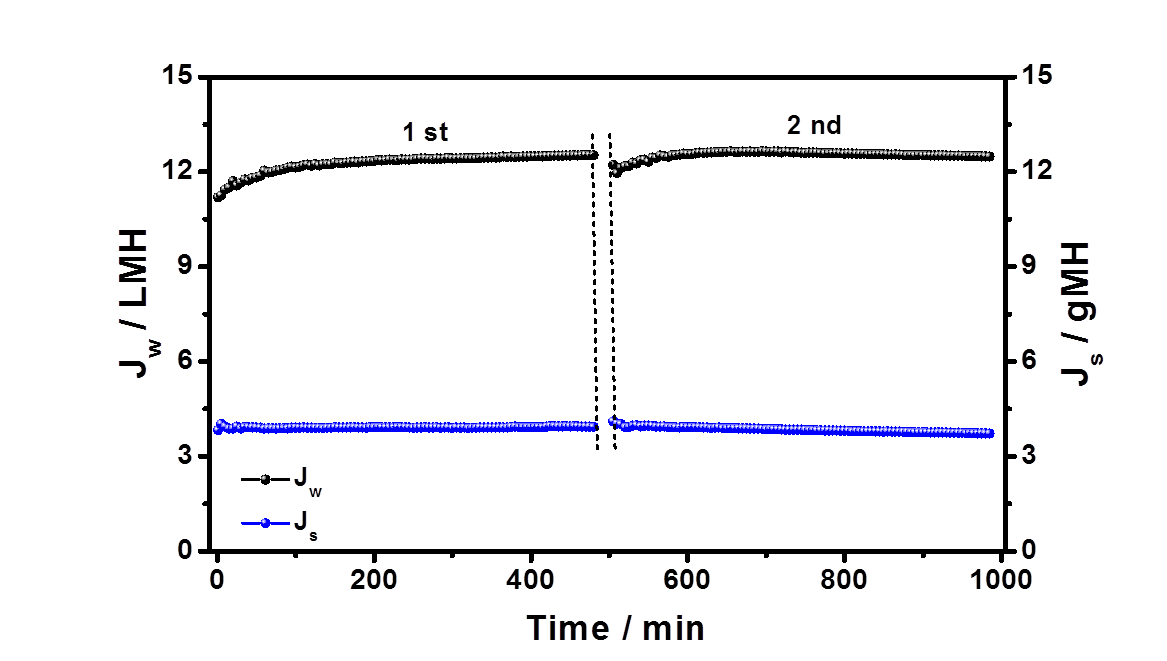


Figure S4. The separation performance of FO-PE coupling system in two consecutive cycle tests.

***
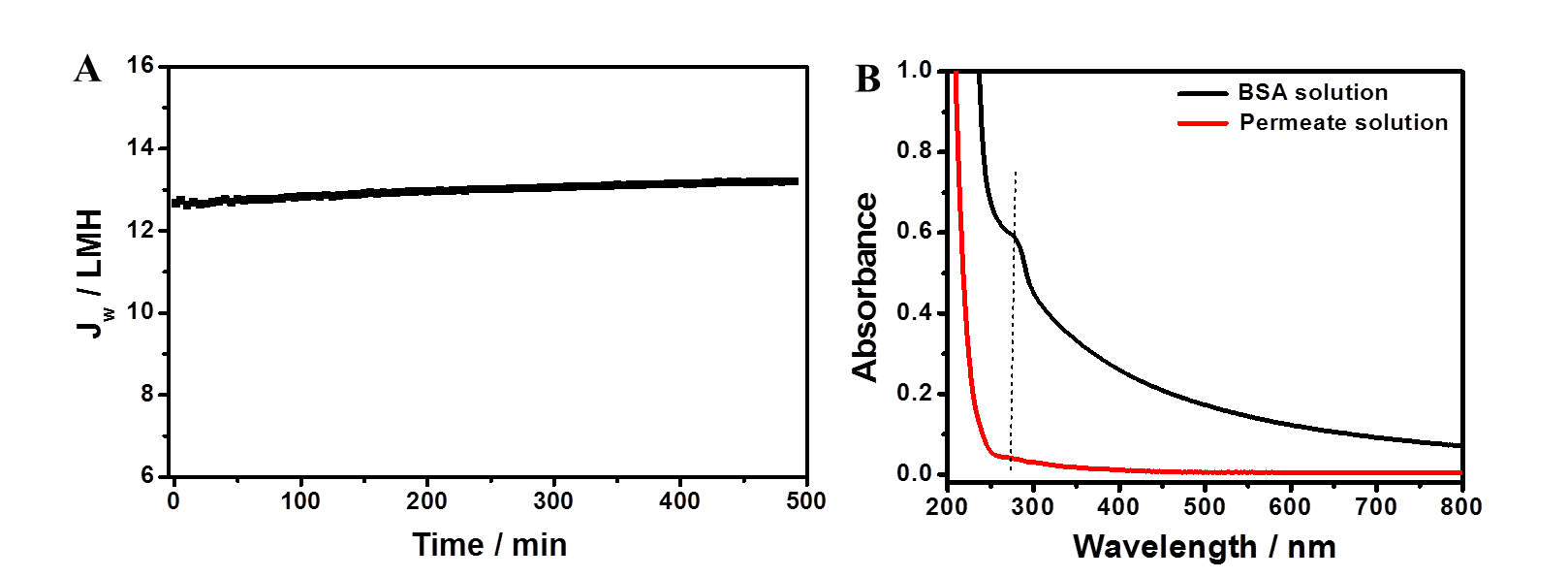
***

*Figure S5. Separation performance of the polyamide FO membrane under FO-PE condition. (A) Time-dependent flux for BSA solution, (B) UV−vis absorption spectra of BSA solution diluted by 4 times (125 mg/L) before and after filtration through polyamide membrane.*
